# Yellow fever control: current epidemiology and vaccination strategies

**DOI:** 10.1186/s40794-020-0101-0

**Published:** 2020-01-10

**Authors:** Lin H. Chen, Mary E. Wilson

**Affiliations:** 1 Mount Auburn Hospital, 330 Mount Auburn Street, Cambridge, MA 02138 USA; 2 Harvard Medical School, Boston, MA USA; 3 Department of Global Health and Population, Harvard T.H. Chan School of Public Health, Boston, MA USA; 4 Department of Epidemiology and Biostatistics, School of Medicine, University of California, San Francisco, USA

**Keywords:** 3–10: yellow fever, Epidemiology, Control, Vaccination, Vector, Dose-sparing, Fractional dosing, Vaccine supply, Outbreak, Flavivirus

## Abstract

Yellow fever (YF) outbreaks continue, have expanded into new areas and threaten large populations in South America and Africa. Predicting where epidemics might occur must take into account local mosquito populations and specific YF virus strain, as well as ecoclimatic conditions, sociopolitical and demographic factors including population size, density, and mobility, and vaccine coverage. Populations of *Aedes aegypti* and *Aedes albopictus* from different regions vary in susceptibility to and capacity to transmit YF virus. YF virus cannot be eliminated today because the virus circulates in animal reservoirs, but human disease could be eliminated with wide use of the vaccine. WHO EYE (Eliminate Yellow Fever Epidemics) is a welcome plan to control YF, with strategies to be carried out from 2017 to 2026: to expand use of YF vaccine, to prevent international spread, and to contain outbreaks rapidly. YF vaccination is the mainstay in controlling YF outbreaks, but global supply is insufficient. Therefore, dose-sparing strategies have been proposed including fractional dosing and intradermal administration. Fractional dosing has been effectively used in outbreak control but currently does not satisfy International Health Regulations; special documentation is needed for international travel. Vector control is another facet in preventing YF outbreaks, and novel methods are being considered and proposed.

## Introduction

Yellow fever (YF) is a mosquito-borne flavivirus that causes outbreaks with high fatality. In the early 1900’s, the Yellow Fever Commission identified mosquitoes as vectors for YF, and mosquito control programs ensued that curtailed YF disease. Today, 47 countries in Africa and Central and South America are considered endemic for YF, and the WHO estimates an annual burden of 200,000 severe cases of YF and up to 60,000 deaths [1]. The main vectors are *Aedes* species, but *Haemagogus* and *Sabethes* are important forest species in South America, and non-human primates are the reservoir.

In 2015 and 2016, Angola and Democratic Republic of the Congo (DRC) experienced large outbreaks, followed by Brazil and Nigeria in 2017 and 2018. Brazil has experienced increased YF outbreaks because an ongoing YF epizootic has expanded endemic zones to areas near the megacities of Rio de Janeiro and Sao Paulo [2]. Alarmingly, unvaccinated travelers visiting endemic areas have acquired YF and died from YF in higher numbers since 2015 compared to the previous several decades [3].

Infection with YF virus can manifest with fever, nausea, vomiting, and abdominal pain. The symptoms may progress in 20% to jaundice, hepatic and renal failure, and bleeding. The case fatality rate from symptomatic YF can reach 50% [4, 5]. The mainstay of YF control involves vector control and YF vaccination.

## Recent yellow fever epidemiology

YF outbreaks continue to occur in Africa and in South America. In Africa, outbreaks affect urban and rural populations. In South America, recent human cases reflect sylvatic transmission – virus circulation among nonhuman primates and spillover into the human population, transmitted by mosquitoes that are found in forested areas (such as *Haemagogus* and *Sabethes* spp). Human infections are largely in males who enter forested areas for work or recreation. Urban transmission by *Aedes aegypti*, the mosquito that now infests cities throughout tropical and subtropical South America, has not been documented in recent outbreaks [2]. The last outbreak of urban YF in Brazil was in 1942.

Virus genomes [6] from the South America outbreak that was recognized in December 2016 provided convincing evidence that infection was due to forest YF with spillover into human populations, leading to > 2000 human cases and > 700 deaths in 2016–2018 in Brazil. Early sylvatic transmission was followed by spatial expansion towards previously YF-free areas. Human cases lagged behind those in nonhuman primates by about 4 weeks. This was the largest epidemic in Brazil in decades. A more recent analysis [7] added other details. Researchers analyzed YF viruses from humans, nonhuman primates, and mosquitoes across 5 Brazilian states (YF endemic and nonendemic) between 2015 and 2018 to reconstruct virus spread. The outbreak in southeastern Brazil, unrecognized until late 2016, originated from a 2014 event in Goias state. The lineage from Goias state was introduced into Minas Gerais at least twice. Virus sub-lineages spread by different routes towards densely populated regions in eastern Brazil. At the start of the epidemic, an estimated 35 million unvaccinated people lived in YF risk areas.

Globally two scenarios cause deep concern: the potential for YF virus to reach the major population centers in eastern Brazil and cause explosive urban outbreaks, and the potential for YF to spread to large densely populated urban centers in Asia. In both places, urban areas are infested with *Aedes aegypti* and/or *Aedes albopictus.* In both areas most people are unvaccinated and susceptible to infection.

## Elements necessary for YF to appear in an area

It is useful to review what elements are necessary for an YF outbreak to occur in a human population. A source of the virus must be present. The virus can potentially be carried into a nonendemic area by a traveler. In many parts of South America the virus circulates in nonhuman primates, which are widely distributed including in urban parks.

A competent mosquito vector must infest an area. The topic of competence is discussed below. The mosquito must have access to a source of the virus (such as an infected human or nonhuman primate). The ecoclimatic conditions, including temperature, rainfall, and humidity, must allow the mosquito to survive long enough for the virus to disseminate in the mosquito to allow onward transmission. The extrinsic incubation period for the virus in the mosquito (the time between taking a blood meal that contains virus until the virus disseminates and can be transmitted via saliva during feeding) is highly dependent on temperature and humidity [8]. In cool areas, the mosquito may die before the virus disseminates and reaches the saliva. The mosquito must then have access to a nonimmune animal or human.

Although YF virus is usually transmitted from viremic host to mosquito to animal or human, another mechanism can maintain the virus. When a female YF virus-infected *Aedes aegypti* mosquito lays eggs (produced in infected ovaries), the mosquitoes that develop from the eggs may carry transmissible YF virus. Vertical transmission (or transovarial transmission) occurs with some other virus-mosquito pairs as well. *Aedes aegypti* eggs are desiccation resistant – they can survive dry conditions. Even if months pass between their production and the next rain, the eggs can still yield viable and infectious progeny. How big a contribution vertical transmission of virus in mosquitoes makes to the overall YF epidemiology is unclear, but it allows virus persistence in the absence of a vertebrate host. Studies by Aitken [9] documented vertical transmission of YF virus in *Aedes aegypti*. More recently Diallo [10] showed vertical transmission of YF virus in two colonies of *Aedes aegypti* from Senegal. The percent of female progeny infected reached 5.2% for those with longer extrinsic incubation periods. Researchers also observed vertical transmission outside the laboratory, finding virus in recently emerged adults from larvae collected in the field.

## Virus mosquito interactions

A complicated biological process occurs within a mosquito that successfully transmits a pathogen, such as a virus, from one host to another. The mosquito must first be attracted to a specific host. Mosquitoes vary greatly in their host preferences. Some, such as *Aedes aegypti*, strongly prefer human blood and will ignore other sources of blood meals if they can find a human. Others, such as *Aedes albopictus*, are more cosmopolitan in preferences and will feed on whatever blood source is available, whether animal or human. *Aedes albopictus* mosquitoes have been responsible for outbreaks of human infections (including dengue and chikungunya) where humans are the main source of blood available. The capacity of *Aedes albopictus* to feed on many different species also means that they can potentially serve as bridge vectors and carry viruses from one species to another, for example, from animals to humans. This attribute, combined with their broader habitat in forested and park areas raises concern that they could transmit viruses usually found in animals to humans.

More than 3000 different species of mosquitoes have been identified globally and > 150 species are found in the US. Most do not transmit human pathogens. Within a species of mosquito, populations are heterogeneous. This means that *Aedes aegypti* mosquitoes that infest Trinidad may not be the same as *Aedes aegypti* mosquitoes that are found in Memphis, Tennessee. Results of laboratory studies done with mosquitoes from one area may not be generalizable to other populations of the same mosquito species.

Viruses are also heterogeneous. YF viruses, which probably originated in Africa, have evolved over time and now are clustered in seven different genotypes, 5 in Africa and 2 in South America. These viruses may differ in important characteristics, such as virulence and transmissibility by specific mosquito populations [11].

## Vector competence and vectorial capacity

Vector competence refers to the ability of a vector, such as a mosquito, to acquire and transmit a pathogen, such as viruses or the malaria parasite [12]. Mosquitoes are refractory to infection by many viruses. A virus must overcome multiple barriers to be transmitted by a mosquito. The virus must infect the epithelial cells of the mosquito midgut. To do this, it must overcome the digestive enzymes in the mosquito, internal microbiota, and the physical barrier of the midgut epithelium. The virus must infect, replicate, be shed and then cross the basal lamina into the hemolymph of the mosquito. To be transmitted, the virus must traverse the basal lamina surrounding the salivary gland and infect acinar cells. From that site, virus can be inoculated into hosts at the time of blood feeding. If the salivary glands are infected, the virus persists in saliva for the life of the mosquito – allowing the mosquito to transmit the virus to more than one host. A virus may infect the midgut but be unable to disseminate or unable to infect the salivary glands. In the laboratory, mosquitoes can be allowed to feed on blood containing viruses and can then be tested at various points in time to assess presence of virus at various sites and its presence in mosquito saliva.

As an interesting aside, the YF vaccine virus (which has been used since 1937) apparently is able to infect mosquitoes but because of the midgut barrier is unable to disseminate in mosquitoes – so it cannot be transmitted by mosquitoes from person to person even though the vaccine virus produces viremia in those vaccinated [11].

It is relevant to know whether a specific vector population is competent to transmit a specific virus. It is even more useful to know the vector efficiency or vectorial capacity. Just because a particular mosquito is competent to transmit a specific virus, does not mean that it can do so efficiently. But even a mosquito that is a relatively inefficient vector, if present in large numbers may be able to sustain an outbreak. Vectorial capacity takes into account the number of mosquitoes relative to the host, the daily blood-feeding rate on the specific host (animal or human), the vector competence (the transmission rate among virus-exposed mosquitoes), the daily survival of the mosquito species or population being studied, and the external incubation period (time it takes for mosquito to transmit virus after initial exposure). Thus the vectorial capacity takes into account environmental as well mosquito factors that affect mosquito behavior, longevity, and biting activity.

Predicting where YF outbreaks might occur is important in planning how to use vaccine but is complicated. To better understand potential for introductions of YF virus into new areas, many laboratory studies have been done to assess specific mosquito populations and their competence to transmit specific YF viruses. Amraoui [13] and colleagues at the Pasteur institute showed that *Aedes albopictus* mosquitoes collected from southeast France could be infected in the laboratory with a West African strain of YF virus. Infectious virus was found in mosquito saliva. Amraoui [14] also studied YF virus and *Aedes albopictus* mosquitoes from Manaus (Brazil). In the laboratory the virus adapted and was excreted in mosquito saliva after 4 passages in *Aedes albopictus*. Couto-Lima et al. [15] showed that *Aedes aegypti* and *Aedes albopictus* as well as forest mosquitoes from YF virus-free areas of Brazil were highly susceptible to American and African strains of YF viruses. This suggests that a traveler returning from YF-endemic parts of Africa could be a source of local transmission in South America. Infestation indexes for *Aedes albopictus* in Brazil are highest in southeastern and southern Brazil where recent outbreaks have occurred. During recent outbreaks in Brazil [6], YF virus was detected in *Aedes albopictus* caught in Minas Gerais in January 2017. An earlier study [16] assessed 23 *Ae aegypti* populations from 13 Brazilian states and found that the mosquitoes were highly susceptible to YF virus. *Aedes aegypti* was eliminated from Brazil in 1955 but control efforts faltered and the country was reinfested in the 1970s. Dengue outbreaks have occurred since the early 1980s and dengue is now endemic in many cities and severe epidemics are common. Earlier Tabachnick [17] tested 28 different populations of *Aedes aegypti* for susceptibility to YF viruses and found extensive variation. Yen [18] showed that *Aedes aegypti* mosquitoes from Guadeloupe (French West Indies) were able to transmit YF virus.

Dengue is endemic and epidemic in wide areas globally that are infested with *Aedes aegypti* and the size and intensity of outbreaks has increased in recent decades. Why is YF virus, which can be transmitted by the same mosquitoes, not similarly widespread in unvaccinated populations? What are the basic differences in the viruses and virus-vector pairs that affect epidemic potential? Both dengue and YF fever viruses produce viremias but the level in YF (10^5^ to 10^6^) [5] is significantly lower than in dengue (can reach 10^7^–10^9^) [19]. Duration of viremia in humans is also longer for dengue than for YF (5 days (3–8) vs. 3 days (2–5)). The basic reproductive number is lower for YF than for dengue [20]. Dengue viruses may also be better adapted than YF viruses to *Aedes aegypti*.

## Models

Many groups have developed models to try to predict populations at risk, anticipate the geographic spread, size of outbreaks, and other findings that may be useful in planning interventions to prevent introductions or control spread.

Brent et al. [21] noted that in 2016, 923 million people lived in areas with endemic YF transmission and 45.2 million travelers departed YF-endemic countries/territories for international destinations. The highest volumes of travelers from YF-endemic countries arrived in Brazil, China, India, Mexico, Peru, and the US. Of note, among those headed for destinations with conditions suitable for transmission, 7.7 million were not required to show proof of YF vaccination upon arrival. Policy changes could help to reduce risk of introductions.

Dorigatti et al. [22] calculated the expected number of YF cases departing from Brazil during incubation or infectious periods during recent outbreaks. They used World Tourism Organization data on volume of air, water, and land border crossings, and suggested that the number of countries that may have received at least one case capable of seeding an epidemic included the US, Argentina, Uruguay, Spain, Italy, and Germany.

A modeling exercise by Shearer [23] found the highest predicted annual case numbers in Nigeria and South Sudan. They predicted high receptivity to transmission in parts of Malaysia, Indonesia, Thailand and noted areas with potential for importation and spread included Central America, eastern Brazil, and SE Asia. They also predicted the relative risk of YF across 47 countries in Americas and Africa. They estimated the number of cases that could be averted by vaccination and provide results for targeted vaccination campaigns.

Kraemer and colleagues [24] using data from recent outbreaks in Angola and Democratic Republic of Congo analyzed datasets for vector suitability, human demography, and mobility to infer district-specific YF infection risk during an epidemic. They provide estimates of the areas that could be prioritized for vaccination. In Africa population density and human movements are important in the spread of YF. Kraemer et al. [25] have also developed maps of the distribution of *Aedes aegypti* and *Aedes albopictus* – past distribution and projections for the future. The size of the populations living in infested areas is projected to increase in the future because of population growth in these regions.

Massad [20] has used models to calculate the critical proportion to vaccinate against YF to prevent epidemic urban YF in a dengue-endemic area. The investigators calculated the force of infection and the relative vector competence for dengue vs. YF viruses. They calculated the basic reproductive number for YF and for dengue for multiple cities in Brazil. The basic reproductive number for YF is lower than for dengue and varies by location for both. The critical percentage needed to be vaccinated to prevent an urban outbreak was as high as 88% in one location.

As a means of controlling YF outbreaks, Massad and colleagues also discussed the possibility of vaccinating monkeys against YF [26] given their role in human outbreaks in Brazil. They discuss feasibility of vaccinating monkeys in smaller green areas in urban centers. In Sao Paulo, where 202 human cases of YF occurred (79 deaths), none was attributed to transmission by *Ae. aegypti*, but most of the urban parks were closed to humans after deaths of monkeys from YF in the parks [27].

## Diagnosis and surveillance

Several authors have emphasized the gaps in surveillance and diagnostic capacity. In both endemic and other countries, lack of good diagnostics can delay recognition of an outbreak. Johansson [28] and colleagues estimated that there may be between 1 and 70 infections that are asymptomatic or mild for every severe case. Early outbreaks may be missed allowing unrecognized spread. In the Democratic Republic of the Congo in 2016, diagnosis of YF was delayed. Most of YF cases had been acquired in Angola. Onset of jaundice, a valuable clinical clue, occurred a week or more after onset of infection [29]. An external quality assessment of European laboratories was conducted in response to the YF outbreak in Brazil and importations by travelers returning to Europe [30]. Adequate capability for diagnosing YF infections was lacking in 10 of 23 countries. Surveillance for YF must include study of nonhuman primates and mosquitoes as well as identification of human infections.

The recent importation of 11 documented cases of YF in Chinese workers from Angola again raises the specter of YFV spread in Asia where massive populations live in areas infested with *Aedes* mosquitoes [31–33]. It is estimated that half a million Chinese travelers have destinations in YF-endemic countries per year [33, 34]. Use of vaccine needs to be expanded and International Health Regulations applied. The imported cases suggest failure of the system to require vaccination for individuals visiting areas of active transmission [32].

Many continue to ponder why YF epidemics have never occurred in Asia. Wasserman and colleagues [35] provide a thoughtful discussion of possible factors. They mention geographical variation in the susceptibility of mosquitoes to YF virus and suggest that wide dengue immunity may play a role. Studies done by Theiler [36] showed that monkeys immunized with pooled human sera from dengue-1 infected volunteers were relatively protected against a challenge with YF virus. Dengue-immune monkeys had lower YF viremia levels. The authors concluded that immunity to dengue may provide a barrier to YF introduction, but this has since been challenged. Dengue virus now circulates in many YF endemic areas in South America and some in Africa.

The Brazil outbreak led to YF being transported to 7 other countries in 2018, including 5 European countries [3]. Chinese working in Angola carried YF back to China. Phylogenetic analysis found the viruses imported to China were homologous with Angola strains [37]. This is the first time that YF has been documented in this region. Increasing travel between Asia and Africa heightens concern about possible outbreaks in Asia [38, 39]. Asian populations are largely nonimmune and unvaccinated except for the tiny fraction who have received YF vaccination before travel.

YF has never emerged in the Pacific despite the presence of competent vectors and warnings of risk [40], especially in the wake of epidemics of chikungunya and Zika virus infections. Populations in the Pacific countries and territories, like Asian countries, have not been immunized against YF. Climate change and warming can potentially make some areas more able to sustain YF virus transmission. Over many decades the distribution of YF in Central America and northern South America shrank in response vaccination campaigns and vector control.

In response to recent outbreaks in Brazil, mass vaccination campaigns have begun. As of late 2018, 13.3 million people in Sao Paulo, 6.5 million in Rio de Janiero, and 1.85 million in Bahia states were vaccinated. Vaccine coverage in those states is now about 55% [41]. In 2018 Bolivia, Brazil, Colombia, French Guiana, and Peru were reporting new YF cases. *Aedes albopictus* mosquitoes naturally infected with YF virus were captured in 2 rural areas in Minas Gerais in 2017. As of April 2019, transmission of YF virus by *Aedes aegypti* had not been documented.

WHO has developed a global strategy to eliminate YF epidemics, EYE, to be carried out from 2017 through 2026 [42]. The three strategic objectives are to protect at-risk populations by expanding use of YF vaccine, to prevent international spread, and to contain outbreaks rapidly. The program will bring together multiple partners; it targets countries and regions most vulnerable to YF outbreaks. Countries are classified taking into account environmental factors, population density, and vector prevalence. Forty countries (27 in Africa and 13 in the Americas) are considered at highest risk. Increased access to YF vaccines is critical. An improved reference genome *Ae aegypti* may also accelerate work on vector control [43].

## YF vaccine

The YF vaccine is a live-attenuated vaccine developed from the wild-type Asibi strain in the 1930s, and passaged in embryonated chicken eggs [1]. All currently available vaccines derive from the substrains 17D-204 (China, France, Senegal, and the US), 17D-213 (Russia), and 17DD (Brazil); 95% of vaccinees become seropositive within 30 days [44]. Four vaccines (Brazil, France, Russia, and Senegal) are WHO prequalified and stockpiled for use in YF vaccination campaigns [1, 44].

Proof of YF vaccination is required for entry into some countries according to the International Health Regulations (IHR). For some travelers visiting endemic countries, YF vaccination is recommended to protect the traveler [4]. The risk of YF illness among travelers to Africa for a 2-week stay is estimated to be 50/100,000 persons and for South America, 5/100,000 persons [4]. Unvaccinated travelers may import the infection to other countries. The concern for YF introduction to susceptible populations arose when persons infected in Angola traveled to/back to DRC, Mauritania, Kenya, and China [44].

Rare severe viscerotropic and neurologic adverse events have caused concern. Estimated rates are 0.3 and 0.8 per 100,000 doses, respectively, and the risk rises for persons aged 60 years and older [4, 45]. Because YF vaccines are live-attenuated, they are contraindicated in immunocompromised persons including persons with immune compromising conditions, those on immune modulating medications, and HIV-infected persons with moderate- to severe immune compromise [4]. The decision about vaccination requires consideration of multiple factors including the traveler’s age, destination, health background, immune status, and whether future travel may benefit from YF vaccination [4, 46–48].

The estimated global YF vaccination coverage from 1970 to 2016 based on sources such as WHO reports and health-service-provider registries that reported YF vaccination activities between May 1, 1939, and Oct 29, 2016 [49] concluded that in order to achieve 80% coverage in YF-endemic populations, between 393·7 to 472·9 million people still need to be vaccinated. This is between 43 and 52% of the population within YF risk zones [49]. The recent high numbers of YF cases was attributed to low vaccination coverage - lower than that needed to prevent outbreaks [23]; it was also estimated that vaccination coverage levels achieved by 2016 averted between 94 336 and 118 500 cases of YF annually within risk zones [23].

## Duration of protection

YF vaccine was previously considered to be valid for 10 years. The WHO Strategic Advisory Group of Experts (SAGE) on Immunization and the World Health Assembly updated the duration to long-term protection, removing the 10-year booster requirement from the IHR [1, 4]. The revision to life-long protection was partly based on the paucity of identified vaccine failures in vaccinated individuals, although post-marketing monitoring for break-through infections is lacking. Since the presence of YF neutralizing antibodies is associated with protection, this recommendation has caused debate about whether a single dose of YF vaccine can protect travelers whose neutralizing antibodies have declined and who are traveling to a high-risk area [50–55].

Lindsey et al. found protective neutralizing antibody levels (PRNT_90_ > 10) following one reported dose of YF vaccine in 146/150 individuals (94%) vaccinated within 10 years (median 4 months, interquartile range [IQR] 2 months–3 years) and 54/66 individuals (82%) vaccinated at 10 years or earlier (median 15 years, IQR 12–25 years) [56]. These findings are comparable to prior studies in vaccinees residing in non-YF-endemic areas [4, 50–52].

There are some concerns regarding subsets of YF vaccine recipients who developed lower antibody responses and/or shorter duration of antibody persistence [4, 50, 51, 57]. In Brazil, seroconversion rates in children were lower when YF vaccine was administered concurrently with measles, mumps, rubella, possibly due to interference from co-administration of these two live-attenuated virus vaccines [58, 59]. Goujon et al. assessed 131 infants; 96% had protective YF antibody levels. All 4 infants without a protective titer of YF antibodies had concurrent MMR and YF administration [59]. YF seropositivity declined in Malian children from 96.7% at 28 days after vaccination to 50.4% at 4.5 years postvaccination; seropositivity also declined in Ghanaian children from 72.7% at 28 days after vaccination to 27.8% at 2.3 years postvaccination [60]. In a nonendemic area, 63.8% of YF vaccinees were seropositive at ≥10 years and seronegativity most likely occured from 3 to 12 years postvaccination [61]. For travelers from non-endemic areas, the findings of blunted response and shorter seroprotection period have led to more conservative recommendations for persons vaccinated during early childhood, during pregnancy, or who were HIV-infected [4]. ACIP also recommends 10-year booster doses for persons who received YF vaccination preceding a hematopoietic stem cell transplant, laboratory workers handling YFV, and travel to higher-risk settings including long stays and travel to areas experiencing outbreaks [4].

## Host concerns

The risk-versus-benefit of immunizing at-risk immunocompromised persons (e.g. HIV-infected, rheumatoid arthritis on disease modifying anti-rheumatic drugs DMARD) against YF vaccine poses challenges. The proliferation of biologic agents and immune modulators has led to the question of whether withholding YF vaccination for immunocompromised travelers is reasonable [62]. Recent studies have been conducted on the safety and immunogenicity of YF vaccination in these groups. Ferreira et al. measured neutralizing antibodies by PRNT and cellular immunity by in vitro YF-specific peripheral blood lymphoproliferative assay [63]. They compared conventional synthetic DMARD (csDMARD) and conventional synthetic plus biological DMARD (cs + bDMARD) to controls and found that only the cs + bDMARD led to an earlier decline in the vaccine response; there was lower PRNT seropositivity between 5 and 9 years and lower effector memory in CD8+ T cells as early as 1–5 years after 17DD-YF vaccination. These finding suggest that a 10-year booster dose of YF vaccine should be administered for persons receiving bDMARD, if they are able to suspend bDMARD [63].

In HIV patients, a study found that patients who received primary YF vaccination while plasma HIV RNA was suppressed maintained high seropositivity 99% within 1 year, 99% at 5 years, and 100% at 10 years [64]. The control of HIV replication at the time of YF vaccination appeared to affect the 10-year immune response; those on successful combination antiretroviral therapy show immune response comparable to that of non-HIV-infected adults up to 10 years [64]. The authors recommend a 10-year YF vaccine booster for patients vaccinated on successful cART, and an early booster for those with uncontrolled HIV RNA.

Finally, 21 human stem cell transplant (HSCT) recipients immunized with YF vaccine at a median of 39 months after HSCT and a median of 33 months after withdrawal of immunosuppression reported no side effects [65]. Eighteen had protective immunity after YF vaccination; furthermore, a third of the recipients who had pre-HSCT YF vaccination had persistent protective immunity after HSCT. If practical, establishing YF seropositivity by measuring antibody titers can help to reassure of immunity in an immunocompromised host.

## Global supply

In YF-endemic countries, WHO recommends that YF vaccine be administered concurrently with the first dose of measles-containing vaccine, and the EYE strategy strives to ensure adequate global vaccine supply. Worldwide there are four WHO-prequalified vaccine manufacturers, and usually there is a stockpile of six million YF vaccine doses to be used if YF epidemics occur, but recent epidemics depleted the stockpile, resulting in the global shortage [66]. The concurrent YF outbreaks in Angola and DRC led to shortage of vaccines and a need for an emergency YF dose-sparing vaccination strategy [67–69]. If YF were to be introduced and spread to other regions, the current global supply of YF vaccine would be insufficient to control outbreaks.

There is only one Food and Drug Administration (FDA)-licensed vaccine in the US, YF-VAX® (Sanofi-Pasteur, Swiftwater, Pennsylvania). In 2017, manufacturing issues resulted in a stockout of YF-VAX® [70]. Sanofi Pasteur worked with the FDA to import another 17D-204 vaccine, Stamaril®, produced in France, in order to supply YF vaccine to US travelers [70]. However, this has led to severe limitations in accessing YF vaccine and significant inconvenience for travelers and providers [71]. Japan experienced a similar YF-VAX® stockout that necessitated the creation of a clinical trial to use Stamaril® [72].

## Dose-sparing strategies and their duration of protection

Two dose-sparing alternatives have been studied: fractional dosing of YF vaccine and intradermal vaccination [67, 68, 73]. The fractional dosing strategy was first used in the large 2016 YF outbreak in the DRC, but excluded pregnant women, children under 2 years of age, and HIV-infected persons [74]. Intradermal YF vaccination has only been used in research setting [73].

Fractional dosing is based on the recognition that the standard vaccine dose, typically contains much higher content of virus (≥10,000 international units (IU)), that exceeds the minimum amount of virus required to achieve a protective titer of neutralizing antibody (1000 IU) [68, 75, 76]. At lower vaccine virus concentrations of 587 IU and lower, there was slower onset of viremia and lower geometric mean titers (GMT), seroconversion rates, and seropositivity at 10 months post-vaccination [75]. However, fractional doses containing ≥587 IU of virus achieved GMTs and seroconversion rates similar to those from full doses, and doses containing ≥3013 IU achieved immune response measurements similar to full doses [75]. The WHO recommended that one-fifth of the 0.5 ml original Brazil-made 17DD vaccine be reconstituted to 0.5 ml and administered subcutaneously to persons aged over 2 years; a full dose was still administered to children aged 9–23 months and pregnant women [1, 68]. Fractional dosing was implemented in the DRC to help control the outbreak and proved to be successful [74]. Seroconversion occurred in 98% of fractional dose recipients at 28 days [74]. de Menezes Martins et al. evaluated immunity to fractional-dose YF vaccine and found 85% to be seropositive 8 years after vaccination [77].

Currently receipt of a fractional dose of YF does not officially meet the IHR requirement. In Canada, YF-VAX® is also the only licensed YF vaccine and fractional dosing is used for international travelers during YF vaccine shortage [78, 79]; CATMAT advised clinicians to document fractional dosing of YF-VAX® in a Certificate of Medical Contraindication to Vaccination provided by the Public Health Agency of Canada [78, 79]. Documentation in this way may ease international travel during periods of YF vaccine shortage. In the US, fractional dose of YF-VAX® is not recommended because efficacy data are still considered limited [70].

Intradermal administration of YF vaccine is another dose-sparing strategy [73, 80, 81]. Previously intradermal administration of reduced influenza vaccine doses demonstrated protective responses that were non-inferior to standard intramuscular vaccination [81]. Intradermal administration offers the theoretical advantage of mimicking the route of natural flavivirus entry [73, 82–84]. For instance, dengue virus is injected by feeding *Aedes* mosquitoes into the skin and infects dendritic cells in the dermis and epidermis. These host immune cells disseminate via lymph to regional nodes, resulting in viremia and systemic infection. YF, another flavivirus, is expected to follow a similar sequence.

The immunogenicity of reduced-dose 17D-YFV (Stamaril, Sanofi Pasteur, France) via intradermal administration (0.1 mL) was non-inferior to that of full-dose subcutaneous administration (0.5 mL) [73, 83]. Follow up of a subgroup at 10 years illustrated 98% had protective YF neutralizing antibodies, also non-inferior to recipients of the standard-dose [83]. Although subject numbers are small, intradermal YF vaccine administration appears to be a feasible option for YF protection.

## Other strategies: new vaccine, primate reservoir, and vector control

Improving YF vaccine supply has led to development of a plant-produced subunit vaccine candidate derived from YF virus envelope protein [85]. While a study found partial protective efficacy in mice, the plant-based vaccine achieved inferior efficacy compared to that of the live attenuated 17DD vaccine [85]. As noted earlier, another strategy that has been suggested is vaccinating monkeys against YF [26].

Vector control has also been a component of YF control strategies. Achee et al. reviewed alternative strategies for YF control [86]. The current vector control strategies include the use of pyrethroid insecticide spraying, larval control including larvicides, insect growth regulators, and bacterial toxins, and biologic agents such as predatory copepods, fish, and Toxorhynchites larvae. Many alternative strategies are being explored [86] (See Table 1).

**Table 1:** Strategies being proposed and explored for vector control [85]

Strategies	Description of methods
Dissemination of larvicidal agents through contaminated adult mosquitoes	Entomopathogenic *Ascomycetes* fungi are potential novel larvicides of dengue vectors, which are also YF vectors
Inhibit mosquito host seeking	Spatial repellents such as transfluthrin and metofluthrin disperse effectively
Traps to reduce vectors	Use traps to remove gravid females
“Attractive toxic sugar baits”	Solutions containing sugar, an attractant, and toxin used indoors and outdoors to kill mosquitoes
Insecticide-treated materials	New technology microencapsulation binds deeper in the fabric and promises increased stability and longer release of the insecticide
“Sterile insect technology”	Release sterilized males with improved sterilization method to reduce offspring population
Release of insects with dominant lethality	Release mosquitoes that carry a transgenic construct that restricts survival, hence reduces that mosquito species
Natural intracellular bacterial symbiont *Wolbachia*	Male mosquitoes infected by *Wolbachia* can reduce the viability of eggs from its female mates
“Gene drives”	A transgenic element inserted into the sequence that leads to mosquito population replacement and population suppression (e.g. by creating a sterile allele)

## Conclusion

Despite the availability of a highly effective vaccine, yellow fever outbreaks have continued and have expanded into new areas in recent years. Many populations remain vulnerable to outbreaks. Increasing global travel and population movements pose risks of introductions into large urban areas in tropical and subtropical areas that are infested with mosquitoes competent to transmit YF. Limited supplies of vaccine have hobbled control efforts. Resources, political will, and leadership will be needed to control YF. Even though the YF virus cannot be eliminated from the animal reservoir today, the tools are available to eliminate YF infections in humans.

## Data Availability

Not applicable.
